# Invisible lines of inequality: intersections of gender, motherhood, and work-based discrimination in Bulgaria

**DOI:** 10.3389/fsoc.2025.1687312

**Published:** 2026-01-05

**Authors:** Lyuba Spasova

**Affiliations:** Institute of Philosophy and Sociology at the Bulgarian Academy of Sciences, Sofia, Bulgaria

**Keywords:** workplace discrimination, gender, motherhood penalty, intersectionality, cumulative disadvantage, Bulgaria

## Abstract

**Introduction:**

Motherhood remains one of the most persistent axes of gender inequality in the labor market. Caregiving responsibilities are linked to pay penalties, stalled career progression, and restricted opportunities, risks intensified in post-socialist Bulgaria by occupational segregation, weak family policy support, and precarious employment. Drawing on intersectionality and cumulative inequality theory, this study investigates how structural inequalities and workplace dynamics intersect to shape experiences of discrimination, distinguishing between general workplace discrimination and bias specifically linked to motherhood.

**Theoretical framework:**

Conceptually, the study uses intersectionality to capture how gender intersects with ethnicity, class, family status, and age to produce distinct disadvantages, and cumulative inequality theory to explain how early exclusions compound over time. We situate these dynamics within gendered organizations—where the “ideal worker” norm and evaluation regimes privilege masculinized availability—and recognize subtle discrimination and cognitive bias as micro-foundations with health and stability consequences. We also consider institutional mechanisms—uneven enforcement, opacity, and weak support—that shape awareness of rights and reporting.

**Methods:**

The analysis is based on nationally representative survey data collected in Bulgaria (December 2022–January 2023) through computer-assisted personal interviews (*N* = 937). The analytic sample included currently or previously employed respondents (*N* = 638) and a subsample of employed mothers (*N* = 345). Dependent variables captured (a) overall personal discrimination and (b) motherhood-related discrimination. Predictors included socio-demographics, employment characteristics, economic strain, health, knowledge of rights, and attitudinal measures. Logistic regressions (bivariate and multivariate) were used to identify significant associations.

**Results:**

In the full working sample, ethnic minority status (OR = 5.72) and economic vulnerability (OR = 3.46) were the strongest predictors of reporting discrimination. Among employed mothers, overall discrimination was associated with economic strain, younger age, and perceptions of unfair hiring. Motherhood-specific discrimination was most strongly predicted by prior personal experience of workplace discrimination (bivariate OR ≈ 15.5; multivariate aOR ≈ 46.2) and younger age, with weaker effects for perceptions of unfair hiring and self-rated health. Ethnicity and education were non-significant within the mothers' subsample, reflecting early-stage exclusion from employment rather than absence of risk.

**Discussion:**

The findings highlight both structural disadvantage and context-specific mechanisms. Early-stage gatekeeping likely filters out the most marginalized before they enter or remain in the category of employed mothers. Cumulative disadvantage is evident, as prior discrimination strongly predicts motherhood-related bias. Age effects suggest younger women face higher risks, potentially linked to precarious employment and employer assumptions about caregiving.

**Conclusion:**

Findings underscore the cumulative and institutional nature of gendered inequality, where early exclusion and organizational norms jointly reproduce disadvantage. Addressing discrimination requires moving beyond formal legislation to tackle organizational cultures, procedural transparency, and early exclusion mechanisms, while strengthening protections for vulnerable groups and supporting employees with caregiving responsibilities.

## 1 Introduction

Motherhood is one of the most persistent axes of gender inequality in the labor market. Caregiving responsibilities are associated with lower pay, stalled career progression, and restricted opportunities—the so-called “motherhood penalty” (Budig and England, 2001; Kalabikhina et al., 2024). These disadvantages are reinforced by organizational norms that privilege uninterrupted careers (Acker, 1990), subtle stereotypes and biases (Reskin, 2000), and structural contexts that shape expectations around gender roles (Cukrowska-Torzewska and Lovasz, 2020; Erlandsson et al., 2023).

Across Europe, the motherhood penalty persists and is shaped by institutional factors. A recent meta-analysis confirms systematic wage losses for mothers, resulting from interruptions and trade-offs with flexibility (Kalabikhina et al., 2024), as well as a father premium and especially large parenthood gaps in Eastern Europe (Cukrowska-Torzewska and Lovasz, 2020). Gatekeeping starts early, with hiring penalties for mothers (Zamberlan et al., 2024) and pregnancy-related discrimination during recruitment and returning to work (Russell et al., 2011). Beyond pay, subtle mechanisms such as microaggressions and bullying are common and have a significant impact on health (Lopes et al., 2023; Sánchez-Sánchez and Fernández Puente, 2024; Pietiläinen et al., 2020). Intersectional patterns—such as compounded barriers for minority women—are clear but under-studied (Martínez-Blancas et al., 2023).

In Bulgaria, persistent pay gaps, occupational segregation, and limited institutional support for work–family reconciliation intensify these risks (Stoilova et al., 2012; Stoilova and Krasteva, 2019). Post-socialist restructuring has deepened inequalities, with precarious employment and minority status amplifying vulnerability (Stoyanova and Kirova, 2008; Luleva, 2024). As in the broader context, there is clear evidence that intersectional patterns—such as compounded barriers for minority women—exist but are under-studied (Martínez-Blancas et al., 2023).

However, research in Central and Eastern Europe, especially Bulgaria, is limited, and even more so is the distinction between general workplace discrimination and bias specifically linked to motherhood. Consequently, there is a lack of evidence on whether the factors influencing discrimination are similar or different across these outcomes and whether sectoral context and life-course cues, such as age as a proxy for caregiving expectations, have varying importance in post-socialist environments.

By integrating intersectionality and cumulative inequality theory within the Bulgarian post-socialist context, the study examines how structural inequalities and workplace dynamics intersect to shape women's experiences of discrimination. By situating the analysis within a post-socialist welfare and labor regime, the study extends intersectionality and cumulative inequality theories to a context where state-socialist legacies, weak family policy, and rapid marketization interact to reproduce gendered risks.

Using nationally representative survey data, we analyze two outcomes: (1) general workplace discrimination among all employed respondents; and (2) motherhood-specific discrimination among employed mothers. We test whether intersectional status (ethnic minority), material vulnerability, organizational levers (sector, position), life-course markers of caregiving expectations (age), institutional capital (knowledge of rights), and strain (self-rated health) differentially predict these outcomes—thereby distinguishing risks that appear broad from those more clearly tied to caregiving.

In doing so, the paper (i) separates general from motherhood-specific discrimination in a CEE setting, (ii) links theory to measurement by operationalizing core mechanisms as observable predictors, and (iii) situates findings in Bulgaria's post-socialist context to offer cautious, context-sensitive implications for sectoral practice, transparency, and rights awareness. To our knowledge, this is the first study to combine nationally representative survey data with an intersectional framework to examine workplace discrimination against mothers in Bulgaria.

## 2 Literature review and theoretical framework

### 2.1 Intersectionality, motherhood penalty, and cumulative disadvantage

Intersectionality (Crenshaw, 1989; Collins and Bilge, 2020; Cho et al., 2013) conceptualizes gender as mutually constituted with other structural locations—such as ethnicity, class, family status, disability, and age—producing compounded disadvantages in labor markets (Martínez-Blancas et al., 2023). A key mechanism is the motherhood penalty, characterized by wage losses, slower progression, and fewer opportunities (Budig and England, 2001; Kalabikhina et al., 2024), often paired with fatherhood premiums (Cukrowska-Torzewska and Lovasz, 2020). Cumulative disadvantage (DiPrete and Eirich, 2006) explains how early-stage filtering (e.g., hiring bias) compounds into durable career gaps (Smith and Waite, 2019; Fernandez-Mateo, 2009), including in elite professions and academia (Halrynjo and Mangset, 2024; Di Leo et al., 2025). Cumulative inequality theory also emphasizes life-course “chain reactions” through resource accrual and risk exposure (Ferraro and Shippee, 2009). Cross-national evidence shows that institutional configurations and normative climates condition the size of motherhood penalties (Grimshaw and Rubery, 2015; Moriconi and Rodríguez-Planas, 2021; Verniers and Vala, 2018; Bygren et al., 2011), with recent CEE evidence of explicit bias against mothers (Faragalla et al., 2023). Pregnancy-related discrimination at recruitment and retention is a key entry point for exclusion (Russell et al., 2011).

### 2.2 Gendered organizations and cultural norms

Acker's (1990) theory shows how organizational routines and the “ideal worker” norm privilege masculinized availability, disadvantaging mothers and minority women. Cultural belief systems translate into differential expectations and evaluations (Ridgeway and Correll, 2004). Organizational change efforts can inadvertently re-embed bias in everyday routines (Ely and Meyerson, 2000), while credentialing and hierarchy shape how discrimination is perceived and enacted (Bobbitt-Zeher, 2011; Mihail and Baroni, 2021). Concepts such as integrated motherhood (Dow, 2016) and whiteness as privilege in STEM (Cech, 2022) specify how class/race structure maternal legitimacy; even with D&I policies, inequality is often reproduced through routine practices (Rodriguez and Guenther, 2022).

### 2.3 Subtle discrimination and cognitive biases

Beyond overt exclusion, inequalities persist via microaggressions, exclusion from decisions, and work-family stereotypes (Lucifora and Vigani, 2016; Lopes et al., 2023). Reskin (2000) links discrimination to automatic categorization and attribution biases that organizations can activate or dampen. Ambivalent sexism (Glick and Fiske, 2001, 2011) legitimizes caregiving ideals while penalizing deviations. Caregiving norms operate as a key axis of exclusion even in progressive cultures (Clark-Saboda and Lemke, 2023), and workplace bullying/harassment functions as structural exclusion across EU labor markets (Sánchez-Sánchez and Fernández Puente, 2024). These processes affect health and job stability (Pietiläinen et al., 2020) and are embedded in broader inequality regimes (Tomaskovic-Devey and Avent-Holt, 2019). These cognitive and attitudinal processes align with the present study's inclusion of perceived unfairness in hiring, witnessed discrimination, and self-rated health as attitudinal and strain-related predictors.

### 2.4 Institutional mechanisms, trust, and statistical discrimination

Awareness, trust, and enforcement gaps blunt formal protections (Kalpazidou Schmidt, 2019), while procedural opacity in pay/promotion reproduces inequality (Castilla, 2015). Evidence from SEE highlights weak and uneven implementation of equality frameworks and policy resistance (Stratigaki, 2012; D'Ancona and Valles Martinez, 2021; Hervías Parejo and Radulović, 2023). Workplace injustice is embedded in power-laden institutions (Roscigno, 2011); migration regimes and national ideologies further structure exclusions, especially for minority and migrant women (Christou and Kofman, 2022; Martiniello and Verhaeghe, 2023; Tahir, 2020). Despite nominal equality, organizations often fail to support care responsibilities (Bird and Brush, 2002). Statistical discrimination based on caregiving assumptions sustains penalties (Dickinson and Oaxaca, 2009; Lesner, 2018), with hiring biases detected in both private and public sectors (Zamberlan et al., 2024; Papamichail et al., 2024). Ethnicity, religion, class and age intersect with gender to shape labor-market sorting and performance evaluations (Diehl et al., 2020; Alon and Haberfeld, 2007; Pedulla, 2014; Jaakson and Dedova, 2023; Nepoti et al., 2025). Institutional awareness and trust indicators operationalize these mechanisms through respondents' self-assessed knowledge of rights and perceptions of fairness.

### 2.5 Bulgarian and regional specificities

In Bulgaria—and across post-socialist Europe—marketisation and limited care infrastructure magnify gendered risks (Glass and Fodor, 2011; Hofäcker et al., 2013; Marra, 2018). Persistent pay gaps are partly attributable to discrimination (Jolliffe, 2001; Stoilova et al., 2012); precarious work and minority status raise exposure (Kirova and Stoyanova, 2005).

Under socialism, women were simultaneously idealized as workers, mothers, and primary caregivers—the so-called “superwoman” ideal that masked a persistent double burden and reinforced patriarchal norms under the guise of equality (Kotzeva, 1999; Daskalova, 2005; Drakulic, 2014). These cultural legacies continued into the post-socialist period, shaping contemporary expectations of women's economic participation and caregiving roles and influencing how discrimination and work–family conflicts are perceived in Bulgaria today (Kotzeva, 1999; Drakulic, 2014).

Post-socialist labor insecurity and minority status amplify vulnerability (Stoyanova and Kirova, 2008). Childcare and pension policy gaps exacerbate mothers' economic disadvantages (Stoilova and Krasteva, 2019; Kingsbury, 2019). Broader social-policy constraints further limit work–family reconciliation (Ivanova and Eneva, 2019). Sectoral case studies document barriers in male-dominated professions (Enchev et al., 2019). Recent ethnographic research highlights how deindustrialization and privatization produced structural uncertainty, reinforcing reliance on gendered family networks that buffer exclusion but also entrench dependency (Luleva, 2024). As a contrast benchmark, even Sweden's high-equality context shows organizational culture and workload constraints on mothers' careers (Erlandsson et al., 2023). These features align with our findings on early-stage gatekeeping, age effects, and the salience of institutional (dis)trust.

### 2.6 This study

Together, these frameworks suggest a multi-level model in which structural inequalities (ethnicity, class, policy) shape access to employment, organizational routines determine exposure to bias, and cumulative disadvantage links past experience to current perceptions.

Building on these theoretical perspectives, the current study analyses two outcomes using nationally representative data: (a) *general discrimination* among all employed respondents; and (b) *motherhood-specific discrimination* among employed mothers. Predictors implement the mechanisms above: intersectional status (ethnic minority), material vulnerability, organizational levers (sector, position), life-course markers of caregiving expectations (age), institutional capital (knowledge of rights), and strain (self-rated health). Guided by the framework, we test:

- Hypothesis 1 (Motherhood-specific): among employed mothers, younger age and employment in the private sector are associated with higher odds of motherhood-linked discrimination, reflecting employer expectations of future caregiving and the “ideal worker” norm (theoretical implications from Sections 2.1, 2.2, and 2.4).
- Hypothesis 2 (General): across all employed respondents, ethnic minority status and material vulnerability predict general discrimination, consistent with intersectional status devaluation and early-stage gatekeeping (theoretical implications from Section 2.1).
- Hypothesis 3 (Sectoral regime): working in the private sector increases the odds of both general and motherhood-specific discrimination, net of controls (theoretical implications from Section 2.2).
- Hypothesis 4 (Strain pathway): poorer self-rated health correlates with higher reports of general discrimination (theoretical implications from Section 2.3).
- Hypothesis 5 (Institutional capital): greater knowledge of anti-discrimination rights is associated with *higher* reporting of general discrimination (recognition/reporting), but may *not* increase motherhood-specific reporting once organizational levers are controlled (theoretical implications from Section 2.4).
- Hypothesis 6 (Hiring/retention filter): younger mothers in the private sector face the steepest odds of motherhood-specific discrimination (sector × age configuration; theoretical implications from Sections 2.2 and 2.4).

## 3 Materials and methods

### 3.1 Study design and data collection

The study draws on nationally representative survey data of adults aged 18 and above, collected in Bulgaria between December 2022 and January 2023 as part of a broader project on risk prevention and management.

The sampling design employs stratified multistage probability sampling (strata: NUTS-3 region × settlement type; PPS selection of primary sampling units; random household and within-household (modified Kish) selection). The sample frame excluded settlements with fewer than 100 residents (about < 1% of the population), and 100 clusters were selected, maintaining the urban–rural distribution. The target sample size of approximately 1,000 was set to achieve 95% confidence intervals with MOE ≈ ±3.2 p.p. under SRS; assuming a design effect of 1.4–1.6, the actual *N* = 937 provides an adequate MOE ≈ ±3.8–4.0 percentage points near 50%. This sample size offers sufficient power for the logistic models, with more than 300 employed mothers meeting standard EPV thresholds. Representativeness is preserved through stratification, PPS, and post-stratification weights (age × sex × region).

Data were gathered through a structured questionnaire administered in face-to-face interviews using computer-assisted personal interviewing (CAPI). The questionnaire covered demographic and socio-economic characteristics, family and household composition, employment and working conditions, perceptions and experiences of discrimination, and awareness of institutional protection mechanisms.

For this study, the analytic sample was restricted to respondents with current or past employment to ensure comparability on work-related discrimination items. After excluding cases with missing data on the dependent variables or key covariates, the final sample size for the full working population was *N* = 638.

Analyses focusing on employed women with children were based on a further restricted subsample defined by affirmative responses to having children and current or past employment (*N* = 345).

### 3.2 Variables and operationalization

#### 3.2.1 Dependent variables

Two outcomes were examined:

- Overall personal discrimination—assessed with the item: “Have you ever personally felt discriminated against?” [Q45: “Някога Вие чувствали ли сте се дискриминиран/а?”] Although positioned in the work-related section of the questionnaire, the question may capture broader experiences. We therefore interpret it as perceived discrimination potentially related to, but not limited to, employment. This outcome was analyzed in both the full working sample and the employed-mothers subsample.
- Motherhood-related discrimination—constructed as a composite “yes” to either of two items asked only to employed women with children: (a) discrimination at work due to frequent childcare-related absences [Q47: “Изпитвали ли сте някога дискриминация на работното място за това, че по-често отсъствате от работа заради децата си?”]; and (b) disadvantage in career advancement due to maternity leave (Q48: “Чувствате ли се ощетена от Вашия работодател относно възможност за професионално израстване заради отсъствието Ви по майчинство?”).

#### 3.2.2 Independent variables

Predictors included:

- Sociodemographics: age (ordered categories), education (low/medium/high), ethnic minority (1) vs. Bulgarian (0), settlement (urban = 1 vs. village = 0).
- Employment: sector (private = 0 vs. public = 1), position (manager/supervisor = 0, worker/employee = 1), job-specialization relation (match = 1 vs. mismatch = 0), adequate qualification (equal/higher = 1 vs. lower = 0), job insecurity (high =1 vs. low = 0).
- Socioeconomic vulnerability**:** inability to cover unexpected expenses (1 = yes; 0 = no), household deprivation index (ordered, 0–7, higher–worse), personal deprivation index (ordered, 0–6, higher–worse), self-assessed material status (good = 1 vs. poor = 0).
- Institutional capital: self-assessed knowledge of anti-discrimination rights, actual legal knowledge index (0–2; higher = more knowledge).
- Health: self-rated health (higher = worse health).
- Attitudes/workplace climate: perceived unfairness in hiring (1 = yes, 0 = no); witnessing discrimination against colleagues (1 = yes, 0 = no), personal discrimination (for motherhood-related discrimination; 1 = yes, 0 = no).
- Composite variables: age^*^sector of employment, ethnic minority^*^household deprivation index, women^*^children (for general discrimination).

### 3.3 Analytical strategy

Logistic regression was selected for the analysis as both outcomes are dichotomous—general discrimination (yes/no) and motherhood-specific discrimination (yes/no)—making a logit link appropriate for modeling event probabilities and producing interpretable odds ratios (and marginal effects) for policy-relevant covariates. Multinomial and ordinal models were considered unnecessary because they would reduce power due to the small number of motherhood cases and introduce additional assumptions (e.g., IIA) without, in this case, offering analytical advantages.

Formally, the model can be expressed as:


$$\begin{array}{l} logit\left(P\left(Y=1\right)\right)\;=\;\beta 0\;+\;\beta 1X1\;+\;\beta 2X2\;+\;\ldots \;+\;\varepsilon . \end{array}$$

The statistical analysis proceeded in four stages:

1. Bivariate logistic regressions on the full working sample (*N* = 638) predicting overall personal discrimination.
2. Bivariate logistic regressions on the employed-mothers subsample (*N* = 345) predicting overall personal discrimination.
3. Bivariate logistic regressions on the employed-mothers subsample (*N* = 345) predicting motherhood-related discrimination.
4. Multivariate logistic regression on the employed-mothers subsample (*N* = 345) predicting motherhood-related discrimination, including all significant bivariate predictors plus theoretically relevant covariates.

The bivariate models (stage1–3) were used to map associations and gather insight for the main models in stage 4. For stage 4 we estimate a hierarchical sequence of models:

- Model 1 (Sociodemographics): age (categorical), education, urban residence, ethnic minority.
- Model 2 (+ Employment): adds sector (public/private), position (manager/supervisor vs. employee), job-qualification relation (match/mismatch), adequate qualification (equal and higher vs. low), and job insecurity (high vs. low).
- Model 3 (+ Resources/Health/Institutional): adds economic vulnerability, deprivation indices and self-assessed material status, self-rated health, knowledge of rights.
- Model 4 (+ Interactions). Adds theoretically motivated interaction terms (e.g., age × sector; minority × deprivation indices) and checks sector-stratified specifications.
- Model 5 (+ Discrimination attitudes/experience). Adds attitudinal indicators about fairness at hiring, witnessed discrimination against colleagues, and overall personal discrimination victimization.

To address selection into the employed-mothers subsample (to correct for differential likelihood of employment among mothers, allowing estimates to reflect population-level trends rather than selection artifacts), we applied inverse-probability weights (IPW) in addition to the survey design weight (in stages 2–4). Propensities for being an employed mother were estimated using a logistic model that included sociodemographics, employment structure (sector, position, job-qualification match) as well as resources/health (economic vulnerability, knowledge of rights, self-rated health) as potential predictors. IPW were trimmed to 0.55–3.34 to limit undue influence. Final analysis weight = survey design weight × trimmed IPW. Post-weighting balance was verified through standardized mean differences, all below 0.10 after weighting, indicating good covariate balance. Standard errors were computed with survey weights; where noted, we confirmed inferences with 5,000 bootstrap resamples (BCa CIs). Given the relatively low prevalence of motherhood-specific discrimination, we employed a hierarchical block strategy and parsimony, reporting confidence intervals and robustness checks rather than relying solely on event-per-variable (EPV) thresholds.

We report adjusted odds ratios (aOR) with 95% confidence intervals; two-sided α = 0.05. Interaction terms are theory-driven; multiple-testing adjustments are not applied to the primary models but interaction findings are interpreted cautiously.

All analyses were conducted in SPSS Statistics (version 31). Statistical significance was set at *P* < 0.05 (two-sided). Model fit was assessed with Cox & Snell *R*^2^, Nagelkerke *R*^2^. We report log-odds (B), standard errors (SE), Wald *P*-values, odds ratios (OR=exp(B)) with 95% CIs, For readability, the Results include a short note on interpreting B, SE, p, and OR. Missing data were handled via listwise deletion within each model; *N* for each analysis is reported in the tables.

### 3.4 Methodological considerations

A limitation of the study design is that it captures self-reported perceptions of discrimination, rather than verified incidents, which may reflect attribution/reporting propensity as much as objective events. This is consistent with our finding that prior discrimination strongly predicts motherhood-specific reports. We therefore interpret results as associations with perceived experiences of discrimination. This distinction between perceived and actual discrimination underlines the interpretive nature of self-reports and reflects what the literature terms “perception bias” (Reskin, 2000).

A further limitation concerns the employed mothers' subsample, which shows restricted variability in certain socio-demographic characteristics. Almost all respondents reporting motherhood-related discrimination were ethnic Bulgarians, and most had medium or high education levels. This homogeneity reduces statistical power and may obscure genuine associations in multivariate models. We therefore treat null results on ethnicity/education in that subsample as inconclusive rather than evidence of no association and explore early-stage selection in the Discussion.

Finally, although the application of inverse-probability and survey weights improves representativeness and reduces selection bias, it cannot fully eliminate compositional differences between employed mothers and the broader working population. Weighted estimates should therefore be interpreted as adjusted for observable characteristics, rather than fully representative of all employed mothers.

## 4 Results

### 4.1 Results from bivariate models

#### 4.1.1 Overall discrimination in the full working sample

In the full working sample (*N* = 638, no IPW weighting), three predictors stood out: ethnic minority status, economic vulnerability, and witnessed discrimination against colleagues (see Table 1). Ethnic minority status was strongly associated with higher odds of reporting discrimination, making it the strongest correlate of reporting personal discrimination (OR = 5.72, *P* < 0.001). Economic vulnerability, measured by inability to afford unexpected expenses, also showed a robust positive association with a significant effect (OR = 3.46, *P* < 0.001). One workplace climate indicator was also positive and robust: reporting discrimination against colleagues was linked to higher odds of reporting personal discrimination (OR = 3.21, *P* < 0.001).

**Table 1:** Bivariate logistic regression—full working/worked sample (*N* = 638) - overall discrimination^a^.

**Predictor**	***B***	**SE**	**Wald**	**df**	***P*-value**	**OR**	**OR_LCL**	**OR_UCL**
Age group 18–29…60+	−0.173	0.097	3.176	1	0.075	0.841	0.696	1.017
Education (Low → High)	−0.76	0.196	14.981	1	0	0.468	0.318	0.687
Ethnic minority (1) vs. Bulgarian (0)	1.744	0.287	36.925	1	0	5.72	3.259	10.039
Settlement: Urban = 1 vs. Village = 0	−0.565	0.335	2.846	1	0.092	0.568	0.295	1.096
Sector: Public vs. Private	0.012	0.002	22.396	1	0	1.012	1.008	1.016
Position: employee vs. manager/supervisor	0.009	0.003	9.977	1	0.002	1.009	1.003	1.015
Job-specialization match (1 = match)	0.009	0.003	9.347	1	0.002	1.009	1.003	1.015
Qualification adequate (1 = equal/higher)	0.009	0.003	8.718	1	0.003	1.009	1.003	1.015
Job insecurity (1 = High 1–3)	0.008	0.002	10.502	1	0.001	1.008	1.004	1.012
Cannot afford unexpected expenses (1 = yes)	1.241	0.243	26.206	1	0	3.459	2.148	5.569
Household deprivation index (0–7; higher = worse)	−0.06	0.054	1.236	1	0.266	0.942	0.847	1.047
Personal deprivation index (0–6; higher = worse)	−0.168	0.065	6.757	1	0.009	0.845	0.744	0.96
Material status: Good(1) vs. Poor(0)	−0.361	0.25	2.095	1	0.148	0.697	0.427	1.138
Self-assessed familiarity with anti-discrimination law	0.493	0.244	4.097	1	0.043	1.637	1.015	2.641
Legal knowledge score 0–2 (Q51)	0.459	0.197	5.415	1	0.02	1.582	1.076	2.328
Self-rated health (higher = worse)	0.239	0.14	2.922	1	0.087	1.27	0.965	1.671
Witnessed discrimination against colleagues	1.167	0.286	16.68	1	0	3.214	1.834	5.627
Perceived unfairness in hiring	0.405	0.242	2.799	1	0.094	1.499	0.933	2.409
Female (1) vs. Male (0)	0.184	0.236	0.611	1	0.434	1.202	0.757	1.909
Has children (1 = yes)	0.34	0.373	0.832	1	0.362	1.405	0.676	2.919
Women × Children (ref = women with children)	0.121	0.237	0.259	1	0.611	1.129	0.709	1.796

^a^Interpretation note: In all logistic models, B indicates the estimated effect in log-odds, SE its precision. Wald and p-values test statistical significance. Statistical significance is conventionally accepted at P < 0.05 (p < 0.001 = highly significant; p < 0.01 = strong significance; p < 0.05 = moderate significance). OR (odds ratio) expresses the relative likelihood of reporting discrimination - OR > 1 indicates higher odds, while OR < 1 indicates a protective effect.

Education exhibited a protective gradient: respondents with higher education had lower odds of reporting discrimination (OR = 0.47, *P* < 0.001).

Two further variables showed moderate positive associations: self-assessed knowledge of anti-discrimination rights (OR = 1.64, *P* = 0.043) and the anti-discrimination knowledge score (0–2; OR = 1.58, *P* = 0.020).

Several structural job indicators reached statistical significance but with trivial magnitudes (OR ≈ 1.01), meaning they lack substantive relevance. Those include sector, position, qualification adequacy, job specialization, and job insecurity.

Predictors not reaching significance (or marginal significance) included material status (OR = 0.70, *P* = 0.148), household deprivation (OR = 0.94, *P* = 0.266), urban/rural location (OR = 0.57, *P* = 0.092), self-rated health (OR = 1.27, *P* = 0.087). Perceived unfairness in hiring trended positive (OR = 1.54) but fell short of significance. Personal deprivation tended to be negative (OR = 0.85) but also fell short of significance.

Gender and parental status were not associated with reporting (women vs. men OR ≈ 1.20, *P* = 0.43; parents vs. non-parents OR ≈ 1.41, *P* = 0.36; women × children, interaction dummy, OR = 1.13, *P* = 0.611.). This aligns with our expectation that motherhood dynamics would not surface on a global discrimination outcome. Accordingly, we probe motherhood-specific discrimination and intersectional interactions (e.g., gender × children, age × sector) in subsequent models.

#### 4.1.2 Working mothers—overall discrimination (bivariate models)

Among employed women with children (*N* = 345, IPW weighted), several predictors were associated with reporting overall personal discrimination in bivariate logistic regressions (see Table 2).

**Table 2:** Bivariate logistic regression—working/worked mothers sample (*N* = 345, IPW weighted)—overall discrimination.

**Predictor**	***B***	**SE**	**Wald**	**df**	***P*-value**	**OR**	**OR_LCL**	**OR_UCL**
Age group 18–29…60+	−0.372	0.12	9.614	1	0.002	0.689	0.545	0.872
Education (Low → High)	−0.281	0.206	1.864	1	0.172	0.755	0.504	1.131
Ethnic minority (1) vs. Bulgarian (0)	1.564	0.37	17.877	1	0	4.778	2.314	9.867
Settlement: Urban = 1 vs. Village = 0	0.224	0.529	0.178	1	0.673	1.251	0.444	3.528
Sector: Public vs. Private	0.008	0.003	6.405	1	0.011	1.008	1.002	1.014
Position: employee vs. manager/supervisor	0.005	0.003	2.119	1	0.145	1.005	0.999	1.011
Job-specialization match (1 = match)	0.005	0.003	2.193	1	0.139	1.005	0.999	1.011
Qualification adequate (1 = equal/higher)	0.004	0.003	2.026	1	0.155	1.004	0.998	1.01
Job insecurity (1 = High 1–3)	0.005	0.003	3.117	1	0.077	1.005	0.999	1.011
Cannot afford unexpected expenses (1 = yes)	0.768	0.308	6.224	1	0.013	2.155	1.179	3.942
Household deprivation index (0–7; higher = worse)	−0.002	0.008	0.048	1	0.826	0.998	0.982	1.014
Personal deprivation index (0–6; higher = worse)	−0.002	0.009	0.039	1	0.843	0.998	0.981	1.016
Material status: Good(1) vs. Poor(0)	−0.329	0.35	0.881	1	0.348	0.72	0.362	1.429
Self-assessed familiarity with anti-discrimination law	−0.015	0.3	0.003	1	0.959	0.985	0.547	1.774
Legal knowledge score 0–2 (Q51)	−0.307	0.351	0.762	1	0.383	0.736	0.37	1.464
Self-rated health (higher = worse)	−0.161	0.169	0.909	1	0.34	0.851	0.611	1.186
Witnessed discrimination against colleagues	0.791	0.389	4.131	1	0.042	2.206	1.029	4.728
Perceived unfairness in hiring	0.657	0.309	4.526	1	0.033	1.929	1.053	3.535

Two predictors showed strong associations. First, belonging to an ethnic minority was linked to substantially higher odds of reporting discrimination (OR = 4.78, *P* < 0.001). Second, age displayed a clear protective gradient: with each step toward an older age category, the odds of reporting discrimination fell by about one-third (OR = 0.69 per category, *P* = 0.002).

Three further predictors showed moderate effects. Mothers who could not afford an unexpected expense had roughly double the odds of reporting discrimination (OR = 2.16, *P* = 0.013). Witnessing discrimination against colleagues was also associated with higher personal reporting (OR = 2.21, *P* =0.042). In addition, perceiving unfairness in hiring was associated with higher reporting (OR = 1.93, *P* = 0.033), the latter two suggesting a spillover awareness effect rather than a protective one.

One result reached statistical significance but was trivial in magnitude: employment sector (public vs. private) had OR ≈ 1.01 (*P* = 0.011) and is not interpreted substantively.

All other tested predictors—education, settlement type, position level, job–specialization match, qualification adequacy, job insecurity, material status, household and personal deprivation indices, self-rated health, and institutional knowledge measures (single item and 0–2 score)—did not show meaningful associations with overall discrimination among working mothers (all *P* ≥ 0.07 and/or ORs close to 1.00).

#### 4.1.3 Working mothers—discrimination because of motherhood/maternity/children (bivariate models)

Among employed mothers (*N* = 345, IPW weighted), motherhood-related discrimination was significantly associated with four strong predictors (see Table 3). First, age shows a strong protective gradient: older mothers are less likely to report discrimination due to children (OR = 0.51; *P* < 0.001 per one category increase). Second, perceived unfairness in hiring is a robust correlate—mothers who believe that some groups are disadvantaged at hiring have nearly threefold higher odds of reporting motherhood-related discrimination (OR = 2.77; *P* = 0.002). Third, overall personal discrimination is, as expected, very strongly associated with motherhood-specific reports (*P* < 0.001; OR ≈ 15.47); because of conceptual overlap, we treat this as a descriptive benchmark rather than a causal driver. Self-rated health is also significant, as better self-rated health is linked to higher reports of motherhood-related discrimination (*P* < 0.001; OR = 0.36).

**Table 3:** Bivariate logistic regression—working mothers sample (*N* = 345, IPW weighted)—motherhood-related discrimination.

**Predictor**	***B***	**SE**	**Wald**	**df**	***P*-value**	**OR**	**OR_LCL**	**OR_UCL**
Age group 18–29..60+	−0.673	0.137	24.036	1	0	0.51	0.39	0.667
Education (Low → High)	0.229	0.218	1.104	1	0.293	1.257	0.82	1.928
Ethnic minority (1) vs. Bulgarian (0)	0.044	0.491	0.008	1	0.928	1.045	0.399	2.736
Settlement: Urban = 1 vs. Village = 0	0.11	0.524	0.044	1	0.834	1.116	0.4	3.118
Sector: Public vs. Private	0.002	0.003	0.324	1	0.569	1.002	0.996	1.008
Position: employee vs. manager/supervisor	0	0.003	0.006	1	0.938	1	0.994	1.006
Job-specialization match (1 = match)	−0.001	0.004	0.123	1	0.726	0.999	0.991	1.007
Qualification adequate (1 = equal/higher)	−0.001	0.004	0.116	1	0.734	0.999	0.991	1.007
Job insecurity (1 = High 1–3)	−0.003	0.003	0.829	1	0.363	0.997	0.991	1.003
Cannot afford unexpected expenses (1 = yes)	0.154	0.303	0.256	1	0.613	1.166	0.644	2.113
Household deprivation index (0–7; higher = worse)	0.046	0.064	0.516	1	0.473	1.047	0.924	1.187
Personal deprivation index (0–6; higher = worse)	0.055	0.074	0.548	1	0.459	1.057	0.914	1.221
Material status: Good(1) vs. Poor(0)	−0.169	0.344	0.24	1	0.624	0.845	0.43	1.657
Self-assessed familiarity with anti-discrimination law	0.163	0.306	0.284	1	0.594	1.177	0.646	2.144
Legal knowledge score 0–2 (Q51)	−0.059	0.329	0.032	1	0.859	0.943	0.495	1.796
Self-rated health (higher = worse)	−1.03	0.226	20.792	1	0	0.357	0.229	0.556
Witnessed discrimination against colleagues	0.427	0.42	1.035	1	0.309	1.533	0.673	3.491
Perceived unfairness in hiring	1.02	0.336	9.225	1	0.002	2.774	1.435	5.358
Personal discrimination victimization (1 = yes)	2.739	0.36	57.895	1	0	15.468	7.64	31.331

No significant associations were observed for ethnicity, education, type of settlement, employment sector, position, job match, adequate qualification, job insecurity, inability to afford unexpected expenses, household and personal deprivation indices, self-assessed material status, perceived familiarity with anti-discrimination rights, or institutional knowledge.

### 4.2 Results from multivariate models

#### 4.2.1 Working mothers—discrimination because of children (multivariate model)

The sequential multivariate model for employed mothers (*N* = 345, IPW weighted) illustrates clearly how the inclusion of successive blocks of covariates modified the pattern of significant predictors (see Table 4). Age consistently remained a protective factor, while in the final model, motherhood-related discrimination was most strongly predicted by prior personal discrimination and perceptions of unfairness in hiring.

**Table 4:** Significant predictors in the sequential logistic regression models.

**Block**	**Significant effects**
Block 1 (Socio-demo)	Age group (1 = 18–29 … 5 = 60+): OR = 0.406 [0.247–0.668], *P* < 0.001
Block 2 (+ Employment)	Age group: OR = 0.345 [0.199–0.598], *P* < 0.001; Job-specialization match (match = 1): OR = 0.147 [0.030–0.711], *P* = 0.017
Block 3 (+ Deprivation/Health/Knowledge)	Age group: OR = 0.411 [0.217–0.779], *P* = 0.006; Job-specialization match (match = 1): OR = 0.091 [0.015–0.533], *P* = 0.008; Self-rated health: OR = 0.335 [0.118–0.948], *P* = 0.039
Block 4 (+ Interactions)	Age group: OR = 0.424 [0.191–0.941], *P* = 0.035; Job-specialization match (match = 1): OR = 0.093 [0.015–0.556], *P* = 0.009; Self-rated health: OR = 0.333 [0.117–0.944], *P* = 0.039
Block 5 (Final: + Attitudes/Experience)	Age group: OR = 0.272 [0.094–0.781], *P* = 0.016; Personal discrimination (self): OR = 46.155 [7.487–284.542], *P* < 0.001; Hiring bias attitude: OR = 4.312 [1.141–16.279], *P* = 0.031
Model summary (Block 5)	*N* = 345 (weighted); −2LL = 84.13; Cox–Snell *R*^2^ =0.318; Nagelkerke *R*^2^ =0.555; classification = 90.7%; sensitivity = 56.0%; specificity = 96.9%

Three predictors remained statistically significant after controlling for all covariates.

First, prior personal experience of discrimination was by far the strongest determinant: mothers who had personally experienced discrimination were almost 46 times more likely to report motherhood-related discrimination (OR = 46.16, *B* = 3.83, *P* < 0.001).

Second, perceived unfairness in hiring was positively associated with reporting motherhood-specific discrimination (OR = 4.31, *B* = 1.46, *P* = 0.031), suggesting that broader perceptions of inequality in recruitment reinforce women's sensitivity to motherhood bias.

Finally, age remained a robust protective factor, with older women significantly less likely to report such experiences (OR = 0.27 per category, *B* = −1.30, *P* = 0.016).

All other predictors—including education, settlement type, ethnic minority status, job-related characteristics, household and personal deprivation indices, and self-rated health—are not statistically significant in the final model. The fact that predictors such as job-specialization mismatch and self-rated health, which reached significance in earlier steps, became non-significant after attitudinal and experiential variables were added, suggests shared variance and possible mediation through the predictors that remained robust. The inclusion of interaction terms (age × sector, minority × deprivation) did not yield additional effects. Overall, the model indicates that motherhood-related discrimination as reported in the study is primarily rooted in prior discriminatory experiences and attitudinal awareness of bias rather than in socioeconomic or job-structural conditions.

### 4.3 Comparative patterns of significant predictors

Across models, some predictors consistently mattered, while others were context-specific. Table 5 compares the predictors of discrimination across models, highlighting how general discrimination among workers is driven primarily by structural disadvantages, while motherhood-specific discrimination as reported by the respondents is shaped by experiential and attitudinal mechanisms.

**Table 5:** Comparative patterns.

**Model/subsample**	**Significant predictors (*P* < 0.05)**	**Interpretation/notes**
Full working sample (*N* = 638)—overall discrimination	Ethnic minority (+); Economic vulnerability (+); Education (–); Anti-discrimination knowledge (single item, +; score, +); Witnessed discrimination (+)	Minority status is the strongest predictor (OR = 5.72); economic hardship, knowledge awareness, and witnessing discrimination increase reporting; education reduces it
Employed mothers—overall discrimination (*N* = 345)	Ethnic minority (+); Economic vulnerability (+); Age (–); Perceived unfairness in hiring (+); Witnessed discrimination (+)	Younger, economically vulnerable mothers and minorities are more likely to report discrimination; perceived unfairness and witnessing discrimination increases reporting
Employed mothers—motherhood-related discrimination (bivariate)	Personal discrimination (+); Perceived unfairness in hiring (+); Age (–); Self-rated health (–)	Motherhood bias is linked to prior discrimination and perceptions of unfair recruitment; older mothers are less likely to report, and healthier mothers are more likely to report
Employed mothers—motherhood-specific discrimination (multivariate)	Personal discrimination (+); Perceived unfairness in hiring (+); Age (–)	After controlling for all covariates, personal discrimination remains the strongest determinant; hiring unfairness increases risk; age is protective
Comparative insight	—	Structural disadvantages (ethnicity, deprivation) and drive general discrimination; motherhood-related bias primarily reflects experiential and attitudinal mechanisms rooted in prior unfair treatment

+, higher odds; –, protective.

## 5 Discussion

This study integrates an intersectional perspective (Crenshaw, 1989; Collins and Bilge, 2020) with theories of gendered organizations (Acker, 1990), statistical discrimination (Dickinson and Oaxaca, 2009), and cumulative disadvantage (DiPrete and Eirich, 2006) to examine discrimination in the Bulgarian labor market, with a particular focus on working mothers. By combining both general and motherhood-specific outcomes, the analysis connects structural inequalities with organizational practices and attitudinal mechanisms.

The findings resonate with comparative European and CEE evidence (Grimshaw and Rubery, 2015; Verniers and Vala, 2018; Bygren et al., 2011; Kalabikhina et al., 2024; Faragalla et al., 2023), showing that the magnitude and mechanisms of discrimination depend on institutional context, care infrastructure, and normative expectations.

Together, the findings reveal both persistent structural inequalities and mechanisms of cumulative awareness and contextual filtering, confirming that discrimination is not only additive but cumulative—rooted in early structural sorting and reinforced by personal experience and organizational culture.

### 5.1 Structural disadvantage and early-stage gatekeeping

In the full working sample, ethnic minority status and economic vulnerability were the strongest predictors of overall discrimination, consistent with intersectional analyses demonstrating how minority positioning and socio-economic precarity amplify exclusion (Martiniello and Verhaeghe, 2023; Stoyanova and Kirova, 2008). Education also showed a protective gradient, indicating that higher social and cultural capital may buffer exposure to unfair treatment. These findings empirically support intersectionality's claim that discrimination is not additive but systemic, operating through overlapping axes of inequality (Collins and Bilge, 2020; Martínez-Blancas et al., 2023).

Among employed mothers, ethnicity and economic vulnerability remained associated with overall discrimination. However, these associations disappeared in the motherhood/maternity specific outcomes, suggesting that structural biases persist, while motherhood-related disadvantages are mediated by other mechanisms—such as caregiving expectations and prior experience.

Taken together, the findings point to structural gatekeeping processes operating before and during employment, consistent with cumulative disadvantage theory (DiPrete and Eirich, 2006; Ferraro and Shippee, 2009): women from minority groups and those with lower education are less likely to enter or remain in the “employed mothers” category. Such early-stage gatekeeping likely occurs during recruitment or retention, filtering the most disadvantaged before they appear in the “employed mothers” category and in later-stage discrimination analyses. This interpretation fits with cross-national and regional evidence showing that pregnancy-related discrimination disadvantages women at the very entry point into employment (Russell et al., 2011) and further compounds over the life course (Smith and Waite, 2019). It also fits with Bulgarian post-socialist studies showing persistent care and policy gaps (Glass and Fodor, 2011; Hofäcker et al., 2013; Stoilova and Krasteva, 2019).

### 5.2 Life-course effects and generational differences

Age emerged as a consistent predictor, with younger women more likely to report discrimination across both general and motherhood-specific outcomes. This age gradient reflects life-course accumulation of disadvantage and also anticipatory bias: younger women are perceived as “potential mothers,” triggering statistical discrimination (Dickinson and Oaxaca, 2009) based on caregiving expectations rather than actual behavior. This aligns with research linking life-course position to inequality exposure (Jaakson and Dedova, 2023) and reinforces Acker's (1990) “ideal worker” framework—where availability and uninterrupted productivity remain normative standards incompatible with caregiving.

This protective age effect invites several possible interpretations. First, it may reflect period changes in workplace culture and state support: younger cohorts entered the labor market during a period of weakened family policy, reduced childcare provision, and intensified labor-market precarity, where discrimination against caregivers has become more visible and acute (Stoilova and Krasteva, 2019; Hofäcker et al., 2013; Luleva, 2024). Second, it may capture heightened awareness among younger women, who are more likely to recognize subtle forms of exclusion as discrimination, whereas older generations often normalized unequal treatment under the cultural ideals of the “superwoman”—simultaneously worker, mother, and primary caregiver—embedded in socialist and post-socialist gender norms (Kotzeva, 1999; Drakulic, 2014; Daskalova, 2005). Third, temporal distance may attenuate recall or salience: older respondents may downplay or forget earlier experiences, especially if these were perceived as part of ordinary gender expectations rather than violations (Roscigno, 2011). Finally, age differences could reflect life-stage reinterpretation—once caregiving demands subside, discrimination related to motherhood becomes less immediate, and thus less likely to be reported (Ferraro and Shippee, 2009; Smith and Waite, 2019). These explanations suggest that lower reporting among older women does not necessarily imply reduced exposure, but rather evolving institutional conditions, generational norms, and subjective frames of recognition.

This finding also parallels cross-national studies (Bygren et al., 2011; Grimshaw and Rubery, 2015) and regional CEE evidence (Faragalla et al., 2023) showing that penalties are particularly pronounced where family-policy supports are weak or employer flexibility is low.

### 5.3 Cumulative disadvantage and the primacy of personal experience

Our strongest finding—that prior personal experience of workplace discrimination predicts motherhood-related bias by an order of magnitude, even after adjusting for socio-demographic and job-related factors, illustrates a classic cumulative inequality process (DiPrete and Eirich, 2006). Early adverse experiences increase vulnerability and awareness, heightening both actual exposure to and recognition of subsequent unfair treatment (heightened vigilance and interpretive readiness). This dynamic suggests that discrimination operates as a self-reinforcing feedback system, linking individual history to organizational bias structures. This association supports cumulative inequality theory (Ferraro and Shippee, 2009), which posits that disadvantages amplify across the life course through both structural and perceptual pathways.

Notably, witnessing discrimination against colleagues did not predict motherhood-related discrimination, underscoring that first-person experiences, not second-hand observation, drive the internalization and reporting of injustice. This finding highlights the psychological and cognitive dimensions of cumulative inequality—what Reskin (2000) terms “automatic discrimination,” where prior exposure shapes interpretive filters.

Together with the age gradient, the result suggests a life-course sequencing: early discrimination in hiring or early tenure produces trajectories that reverberate through the motherhood stage, reinforcing stratification within gender categories.

This interpretation aligns with meta-analytic and comparative research documenting persistent motherhood penalties but varying institutional intensities (Kalabikhina et al., 2024; Di Leo et al., 2025; Halrynjo and Mangset, 2024).

### 5.4 Workplace climate and attitudinal mechanisms

Perceptions of unfair recruitment practices were positively associated with both general and motherhood-specific discrimination in bivariate models, and remained directionally consistent in the multivariate model, indicating that perceived unfairness operates both as an experiential correlate and as a cognitive filter shaping sensitivity to discrimination. These attitudinal predictors confirm gendered organizations theory (Acker, 1990), which emphasizes that informal norms and opaque procedures, rather than formal policy, sustain inequality. They also illustrate how statistical discrimination is reproduced through organizational expectations about availability and caregiving (Ridgeway and Correll, 2004; Lesner, 2018).

Such perceptions likely reflect Reskin's (2000) subtle bias processes and “automatic discrimination,” where organizational routines activate categorization biases, and connect directly to the statistical-discrimination framework. Such mechanisms are corroborated by factorial survey research showing that recruiters themselves systematically disadvantage mothers in hiring decisions (Zamberlan et al., 2024). This convergence between perceived and experimentally demonstrated bias underscores the organizational embeddedness of discrimination across Europe (Castilla, 2015).

Although sector did not emerge as a robust predictor, this non-significance is itself telling of the weak institutional differentiation between public and private employers in Bulgaria (see Hofäcker et al., 2013; Ivanova and Eneva, 2019), consistent with evidence of limited public–private divergence in work-family supports in parts of CEE.

Across models, institutional awareness appears as an enabling rather than protective factor: respondents more familiar with their rights are more likely to recognize and report discrimination. This aligns with Kalpazidou Schmidt (2019) and Roscigno (2011), highlighting that legal consciousness amplifies perceived inequality even when formal protections exist.

### 5.5 Health, vulnerability, and bidirectional risks

The negative association between self-rated health (where higher scores indicate poorer health) and motherhood-related discrimination suggests a potential feedback loop between bias and wellbeing (Pietiläinen et al., 2020), implying that women in better health are more likely to report discrimination—possibly because they are more active, visible, or assertive within organizations and thus more aware of bias. Conversely, those in poorer health may experience lower participation or underreporting due to withdrawal or reduced exposure.

While this pattern diverges from the expected strain pathway linking discrimination, stress, and wellbeing (Pietiläinen et al., 2020; Sánchez-Sánchez and Fernández Puente, 2024), it nonetheless underscores the reciprocal link between wellbeing, agency, and perceived discrimination. Health should therefore be interpreted not as a simple vulnerability marker, but as part of the broader interplay between individual capacity, organizational climate, agency, and labor-market embeddedness.

### 5.6 Implications for policy and practice

The findings indicate that interventions must extend beyond formal anti-discrimination laws to address structural, organizational and cultural factors:

- Organizational level: enhance transparency in recruitment and promotion (Castilla, 2015), normalize flexible arrangements for caregiving employees, and address subtle workplace climate issues.
- Policy level: strengthen institutional trust and enforcement (Kalpazidou Schmidt, 2019), expand protections for economically vulnerable and minority workers, and reduce disparities before they become entrenched.
- Preventive measures: target early-stage gatekeeping to ensure women from minority and lower-education backgrounds are not excluded at hiring or retention stages (Russell et al., 2011).
- Support structures: expand training, awareness campaigns, and accessible reporting mechanisms to reduce both overt and subtle bias. Policies must also tackle overt forms of discrimination that remain prevalent in Bulgarian workplaces, such as sexual harassment and gender-based exclusion, which persist alongside more normalized practices (Ivanova and Eneva, 2019; Tomaskovic-Devey and Avent-Holt, 2019; Lucifora and Vigani, 2016; Lopes et al., 2023).

Such multi-level strategies align with intersectional and gendered-organization theories, emphasizing that equality requires restructuring both opportunity regimes and cultural norms.

### 5.7 Limitations and directions for future research

The cross-sectional design limits causal inference, and the measure of “overall discrimination,” though placed in a work-related section, was not explicitly restricted to workplace incidents. Further, our outcomes capture self-reported perceptions of discrimination rather than verified incidents. Although IPW weighting improves representativeness, it does not fully eliminate sample-selection bias, particularly in the smaller motherhood subsample. That is why the limited variability of ethnicity and education in the employed-mothers subsample constrains statistical power and reflects substantive early-stage exclusion processes.

Future research should employ larger, more socio-demographically diverse samples, ideally with longitudinal data, to capture cumulative effects over time and clarify the relationship between health and discrimination. Qualitative studies could further illuminate how organizational climate, life-course position, and early career experiences shape discrimination trajectories. Triangulation with administrative complaints or audit/vignette evidence might better separate perception from occurrence. Because prior discrimination may reflect both real exposure and heightened sensitivity, future longitudinal designs should disentangle causality from awareness effects.

## 6 Conclusion

The analysis demonstrates that both structural inequalities and context-specific mechanisms shape discrimination against working mothers in Bulgaria. Three key insights emerge.

First, in the general working population, ethnic minority status and economic vulnerability emerged as the strongest correlates of reporting discrimination, confirming the intersectional pattern of structural disadvantage and economic precarity.

Second, within the group of working/worked mothers, age and prior personal experience of discrimination are the most powerful predictors of motherhood-related bias, confirming the cumulative nature of disadvantage: earlier negative experiences increase both exposure to and recognition of subsequent bias.

Third, the disappearance of ethnic and economic effects in the mothers' subsample points to early-stage exclusion—that is, women most exposed to structural risk may be filtered out of stable employment long before motherhood-related barriers become visible.

Together, these findings support the notion of discrimination as a multi-stage and cumulative process, shaped by both early selection and ongoing organizational dynamics.

To address these intertwined mechanisms, policies must also account for the interplay between economic vulnerability, health, and discrimination, recognizing that risks are shaped as much by structural position as by workplace practices. Tackling discrimination requires structural, cultural, and organizational reforms that move beyond formal legal protections.

## Data Availability

The dataset used in this study is not publicly available due to ethical and legal restrictions related to participant confidentiality and data protection. Access may be granted upon reasonable request to the corresponding author and with permission from the data-collecting institution. Requests to access the datasets should be directed to: lyuba.spasova@ips.bas.bg.
